# Supplementary material for: Molecular analysis and essentiality of Aro1 shikimate biosynthesis multi-enzyme in Candida albicans
Source: Life Sci Alliance. 2022 May 5;5(8):e202101358. doi: 10.26508/lsa.202101358 (PMC9074039; doi:10.26508/lsa.202101358)
Supplement: Supplementary file 1 [file LSA-2021-01358_TableS1.docx]

**Table S1. X-ray crystallographic statistics.**

| Structure | Aro1_DHQS_ | Aro1_EPSPS_ | Aro1_SK+DHQase+DHSD_ |
| --- | --- | --- | --- |
| PDB code | 6C5C | 7TBU | 7TBV |
| *Data collection* |  |  |  |
| Space group | P2_1_ | P2_1_ | P2_1_ |
| Unit cell  a, b, c (Å)  α, β, γ, (°) | 50.68, 92.06, 88.92  90, 97.6, 90 | 44.99, 159.75, 55.87  90, 92.3, 90 | 77.68, 89.24, 270.71  90, 90.28, 90 |
| Resolution, Å | 33.00 – 1.85 | 30.00 – 1.85 | 30.0 – 2.30 |
| R_merge_^a^  R_pim_ | 0.045 (0.956)^*^  0.021 (0.595) | 0.084 (1.191)  0.041 (0.589) | 0.154 (1.411)  0.063 (0.595) |
| CC_1/2_^*^ | 0.717 | 0.502 | 0.656 |
| *I* / σ (*I*) | 37.8 (1.44) | 18.10 (1.27) | 13.96 (1.41) |
| Completeness, % | 99.9 (100) | 100 (100) | 99.9 (99.5) |
| Redundancy | 4.8 (3.5) | 5.1 (5.0) | 6.8 (6.5) |
| *Refinement* |  |  |  |
| Resolution, Å | 30.00 – 1.85 | 29.66 – 1.85 | 29.82 – 2.30 |
| No. unique reflections:  working, test | 66446, 2108 | 64723, 1939 | 157517, 1926 |
| R-factor/free R­-factor^b^ | 17.6/20.3 (30.7/31.5) | 18.0/22.9 (27.6/31.6) | 17.8/22.6 (29.5/35.6) |
| No. refined atoms, molecules  Protein  Co-factor  Substrate  Solvent  Water | 5948, 2  88, 2  N/A  53  389 | 6609, 2  N/A  32, 1  16  826 | 21347, 4  N/A  N/A  84  1949 |
| B-factors  Protein  Co-factor  Substrate  Solvent  Water | 42.0  33.5  N/A  47.8  43.5 | 38.07  N/A  36.9  42.0  43.6 | 53.1  N/A  N/A  62.6  50.2 |
| r.m.s.d.  Bond lengths, Å  Bond angles, ° | 0.014  1.655 | 0.007  0.975 | 0.003  0.549 |

*All values in brackets and CC_1/2_ values refer to highest resolution shells.

^a^*R*_merge_ = Σ_hkl_Σ_j_|*I*_hkl.j_ - ​​〈*I*_hkl_〉|/Σ_hkl_Σ_j_*I*_hk,j_, where *I*_hkl,j_ and〈*I*_hk,j_〉are the *j*th and mean measurement of the intensity of reflection *j*.

^b^*R*_pim_ = Σ_hkl_√(n/n-1) Σ^n^_j=1_|*I*_hkl.j_ - 〈*I*_hkl_〉|/Σ_hkl_Σ_j_I_hk,j_

^c^Value refers to highest resolution shell

^d^*R* = Σ|F_p_^obs^ – F_p_^calc^|/ΣF_p_^obs^, where F_p_^obs^ and F_p_^calc^ are the observed and calculated structure factor amplitudes, respectively.

N/A = not applicable.
