# Supplementary material for: Molecular analysis and essentiality of Aro1 shikimate biosynthesis multi-enzyme in Candida albicans
Source: Life Sci Alliance. 2022 May 5;5(8):e202101358. doi: 10.26508/lsa.202101358 (PMC9074039; doi:10.26508/lsa.202101358)
Supplement: Supplementary file 2 [file LSA-2021-01358_TableS2.docx]

**Table S2. Cryo-EM data collection, refinement, and validation statistics**

|  | Aro1 full-length homogeneous refinement  EMDB: 26357  PDB: 7U5S | Aro1_DHQS-EPSPS_  local refinement  EMDB: 26358  PDB: 7U5T | Aro1_SK-DHQase-DHSD_ local refinement  EMDB: 26359  PDB: 7U5U |
| --- | --- | --- | --- |
| **Data Collection and Processing** |  |  |  |
| Magnification | 105,000 | | |
| Voltage (kV) | 300 | | |
| Electron exposure (e-/Å^2^) | 83.5, 72.4 | | |
| Defocus range (µm) | -1.5 to -3.0 | | |
| Symmetry imposed | C1 | | |
| Initial particle number | 1,312,555 | | |
| Final particle number | 87,484 | | |
| Map resolution (Å) | 4.19 | 3.43 | 3.16 |
| FSC threshold | 0.143 | 0.143 | 0.143 |
| **Refinement** | | | |
| Model resolution (Å) | 4.6 | 3.5 | 3.2 |
| FSC threshold | 0.143 | 0.143 | 0.143 |
| Model composition | | | |
| Non-hydrogen atoms | 23,086 | 9,093 | 10,035 |
| Protein residues | 3,006 | 1,190 | 1,283 |
| R.M.S deviations | | | |
| Bond lengths (Å) | 0.008 | 0.008 | 0.007 |
| Bond angles (°) | 1.020 | 1.084 | 0.877 |
| Validation | | | |
| MolProbity score | 1.89 | 1.84 | 1.65 |
| Clashscore | 9.12 | 7.60 | 5.12 |
| Poor rotamers (%) | 0.00 | 0.10 | 0.00 |
| Ramachandran plot | | | |
| Favored (%) | 94.00 | 93.60 | 94.48 |
| Allowed (%) | 6.00 | 6.40 | 5.52 |
| Disallowed (%) | 0.00 | 0.00 | 0.00 |
