# Supplementary material for: Molecular analysis and essentiality of Aro1 shikimate biosynthesis multi-enzyme in Candida albicans
Source: Life Sci Alliance. 2022 May 5;5(8):e202101358. doi: 10.26508/lsa.202101358 (PMC9074039; doi:10.26508/lsa.202101358)
Supplement: Supplementary file 3 [file LSA-2021-01358_TableS3.docx]

**Table S3. Strains used in this work.**

| **Strain** | **Genotype** | **Source or Reference** |
| --- | --- | --- |
| SC5314 | *Candida albicans.* Prototrophic. | (Jones, *et al.*, 2004) |
| SN95 | *Candida albicans* *arg4 /arg4 his1/his1 URA3/ura3::imm434 IRO1/iro1::imm434* | (Noble & Johnson, 2005) |
| CaSS1 | *Candida albicans his3::hisG/his3::hisG leu2::tetR-GAL4AD-URA3/LEU2* | (Roemer, *et al.*, 2003) |
| CaLC5759 | As SN95, *SAT1-TAR-tetO-ARO1/ARO1* | This work |
| CaLC5761 | As SN95, *SAT1-TAR-tetO-ARO1/SAT1-TAR-tetO-ARO1* | This work |
| CaLC6830 | As SN95, *SAT1-TAR-tetO-His_6_TEVARO1/SAT1-TAR-tetO-His_6_TEVARO1* | This work |
| CaLC7800 | *As SN95, SAT1-TAR-tetO-DQD1/DQD1* | This work |
| CaLC7801 | *As SN95, TAR-tetO-DQD1/DQD1* | This work |
| CaLC7803 | *As SN95, TAR-tetO-DQD1/ SAT1-TAR-tetO-DQD1* | This work |
| CaLC6598 | As CaLC5759, *SAT1-TAR-tetO-ARO1/P_ACT1_-His_6_TEVARO1-ARG4* | This work |
| CaLC7016 | As CaLC6598, *SAT1-TAR-tetO-ARO1/P_ACT1_-His_6_TEVARO1^H264K^-ARG4* | This work |
| CaLC7205 | As CaLC6598, *SAT1-TAR-tetO-ARO1/P_ACT1_-His_6_TEVARO1^H280K^-ARG4* | This work |
| CaLC7925 | As CaLC6598, *SAT1-TAR-tetO-ARO1/P_ACT1_-His_6_TEVARO1^D715A^-ARG4* | This work |
| CaLC7212 | As CaLC6598, *SAT1-TAR-tetO-ARO1/P_ACT1_-His_6_TEVARO1^H796A^-ARG4* | This work |
| CaLC6844 | As CaLC6598, *SAT1-TAR-tetO-ARO1/P_ACT1_-His_6_TEVARO1^W877K^-ARG4* | This work |
| CaLC6846 | As CaLC6598, *SAT1-TAR-tetO-ARO1/P_ACT1_-His_6_TEVARO1^S880K^-ARG4* | This work |
| CaLC7281 | As CaLC6598, *SAT1-TAR-tetO-ARO1/P_ACT1_-His_6_TEVARO1^D890A^-ARG4* | This work |
| CaLC7207 | As CaLC6598, *SAT1-TAR-tetO-ARO1/P_ACT1_-His_6_TEVARO1^R980A^-ARG4* | This work |
| CaLC7018 | As CaLC6598, *SAT1-TAR-tetO-ARO1/P_ACT1_-His_6_TEVARO1^R1021E^-ARG4* | This work |
| CaLC7235 | As CaLC6598, *SAT1-TAR-tetO-ARO1/P_ACT1_-His_6_TEVARO1^R1022E^-ARG4* | This work |
| CaLC6840 | As CaLC6598, *SAT1-TAR-tetO-ARO1/P_ACT1_-His_6_TEVARO1^E1072R^-ARG4* | This work |
| CaLC6614 | As CaLC6598, *SAT1-TAR-tetO-ARO1/P_ACT1_-His_6_TEVARO1^R1194E^-ARG4* | This work |
| CaLC7210 | As CaLC6598, *SAT1-TAR-tetO-ARO1/P_ACT1_-His_6_TEVARO1^K1334E^-ARG4* | This work |
| CaLC7208 | As CaLC6598, *SAT1-TAR-tetO-ARO1/P_ACT1_-His_6_TEVARO1^D1370A^-ARG4* | This work |
| CaLC6618 | As CaLC6598, *SAT1-TAR-tetO-ARO1/P_ACT1_-His_6_TEVARO1^P1384EP1385E^-ARG4* | This work |
| CaLC7020 | As CaLC6598, *SAT1-TAR-tetO-ARO1/P_ACT1_-His_6_TEVARO1^G1487E^-ARG4* | This work |
| CaLC6599 | As CaLC6598, *SAT1-TAR-tetO-ARO1/P_ACT1_-His_6_TEVARO1^DHQS^*^Δ^*-ARG4* | This work |
| CaLC6842 | As CaLC6598, *SAT1-TAR-tetO-ARO1/P_ACT1_-His_6_TEVARO1^EPSPS^*^Δ::(GSS)6^*-ARG4* | This work |
| CaLC7209 | As CaLC6598, *SAT1-TAR-tetO-ARO1/P_ACT1_-His_6_TEVARO1^SK^*^Δ::(GSS)6^*-ARG4* | This work |
| CaLC7014 | As CaLC6598, *SAT1-TAR-tetO-ARO1/P_ACT1_-His_6_TEVARO1^DHQase^*^Δ::(GSS)6^*-ARG4* | This work |
| CaLC6615 | As CaLC6598, *SAT1-TAR-tetO-ARO1/P_ACT1_-His_6_TEVARO1^DHSD^*^Δ^*-ARG4* | This work |
