# Supplementary material for: Molecular analysis and essentiality of Aro1 shikimate biosynthesis multi-enzyme in Candida albicans
Source: Life Sci Alliance. 2022 May 5;5(8):e202101358. doi: 10.26508/lsa.202101358 (PMC9074039; doi:10.26508/lsa.202101358)
Supplement: Supplementary file 4 [file LSA-2021-01358_TableS4.docx]

**Table S4. Plasmids used in this study.**

| **Plasmid** | **Description** | **Reference or source** |
| --- | --- | --- |
| pBAD24 | Plasmid vector with L-arabinose-inducible promoter; Amp^R^ | (Guzman, *et al.,* 1995) |
| pLC576 | Plasmid vector encoding the *ARG4* selectable marker; Amp^R^ | (Lavoie, *et al.*, 2008) |
| pLC605 | Plasmid vector encoding a tetracycline-repressible transactivator (*TAR*), frt-flanked *SAT1-P_SAP2_-FLP* recyclable selectable marker, and tetO promoter; Amp^R^, NAT^R^ | (Veri, *et al.*, 2018) |
| pLC620 | Plasmid vector encoding the ACT1 promoter; Amp^R^ | (Shapiro, *et al.*, 2012) |
| pLC963 | plasmid encoding a solo system *Ca*Cas9/sgRNA entry expression vector; Amp^R^, NAT^R^ | (Veri, *et al.*, 2018) |
| pLC1335 | pBAD24 derivative encoding *C. albicans* *ARO1* with an N-terminal TEV protease-cleaveable hexahistidine tag; Amp^R^ | This work |
| pLC1349 | pBAD24 derivative encoding *C. albicans* *ARO1* with an N-terminal TEV protease-cleaveable hexahistidine tag; Amp^R^ | This work |
