# Supplementary material for: Molecular analysis and essentiality of Aro1 shikimate biosynthesis multi-enzyme in Candida albicans
Source: Life Sci Alliance. 2022 May 5;5(8):e202101358. doi: 10.26508/lsa.202101358 (PMC9074039; doi:10.26508/lsa.202101358)
Supplement: Supplementary file 5 [file LSA-2021-01358_TableS5.docx]

**Table S5. Oligonucleotide primers used in this study.**

| **Primer** | **Description** | **Sequence (5ʹ-3ʹ)** |
| --- | --- | --- |
| oLC5979 | sgRNA cassette *P_ARO1_*-Fw | CCGCaagtgattagacttag |
| oLC5981 | sgRNA cassette *P_ARO1_*-Rv | GGTGGCGGCAAAACTAATTC |
| oLC6924 | *CaCAS9*-Fw | ATCTCATTAGATTTGGAACTTGTGGGTT |
| oLC6925 | *CaCAS9*-Rv | TTCGAGCGTCCCAAAACCTTCT |
| oLC7047 | *P_ARO1_*-sgRNA-Fw | TAAAAACTAAGACATACTGGGTTTTAGAGCTAGAAATAGC |
| oLC7048 | *P_ARO1_*-sgRNA-Rv | CCAGTATGTCTTAGTTTTTACAAATTAAAAATAGTTTACG |
| oLC7059 | *tetO-CaARO1*-Fw | TAGAATAAATTGAGAGTTTTTTGTTTCCCAACACCCCAATATCTAGGTATAACAGTTTATGAACAGCATAAGggaaacagctatgaccat |
| oLC7060 | *tetO-CaARO1*-Rv | TATGGTCGGCAATACCATAACCAACATGGATAGTTTCCTTACCCAAAATTGGCACCTTTTCAATAGACATcgactatttatatttgtatg |
| oLC7071 | *ARO1-*out-Fw | TACTGTTCGGGTCACAGGTG |
| oLC7072 | *ARO1-*out-Rv | CTTCTATTCAATCGACATACG |
| oLC7073 | *ARO1-*out-Fw-2 | GTATGGCAATTAGATAATCCTC |
| oLC7074 | *ARO1-*in-Rv | GGAACTTGAACAACTCTGAC |
| oLC7075 | *ARO1-*in-Fw | CAATCTGCTGGTTTCAAGCC |
| oLC7076 | *ARO1-*in-Fw-2 | CCAATTTCTCAATTATACAT |
| oLC7077 | *ARO1-*In-Rv-2 | GAGGAAGAACCTATGTTCGG |
| oLC7108 | *CaARO1*H264K-SDM-Fw | CTTAGAAATTTACTTAATTTTGGCaaaACTATTGGTCATGCAATTGAAGCGG |
| oLC7109 | *CaARO1*H264K-SDM-Rv | CCGCTTCAATTGCATGACCAATAGTtttGCCAAAATTAAGTAAATTTCTAAG |
| oLC7110 | *CaARO1*H280K-SDM-Fw | GGTTTTAACTCCAGAAGCATTAaaaGGAGAATGTGTTTCCATTGG |
| oLC7111 | *CaARO1*H280K-SDM-Rv | CCAATGGAAACACATTCTCCtttTAATGCTTCTGGAGTTAAAACC |
| oLC7112 | *CaARO1*D715A-SDM-Fw | TTGATATGGAGCCAATGACTgctGCATTTTTGACTGCTTCTGTTG |
| oLC7113 | *CaARO1*D715A-SDM-Rv | CAACAGAAGCAGTCAAAAATGCagcAGTCATTGGCTCCATATCAA |
| oLC7114 | *CaARO1*H796A-SDM-Fw | GTGTTTCCTCCTATGATGATgctAGAGTGGCTATGTCATTTTC |
| oLC7115 | *CaARO1*H796A-SDM-Rv | GAAAATGACATAGCCACTCTagcATCATCATAGGAGGAAACAC |
| oLC7116 | *CaARO1*D890A-SDM-Fw | GGGGTTCAAGATGTTAGATATGgctAAATATCTTGAAGAGAAATTGG |
| oLC7117 | *CaARO1*D890A-SDM-Rv | CCAATTTCTCTTCAAGATATTTagcCATATCTAACATCTTGAACCCC |
| oLC7118 | *CaARO1*R980A-SDM-Fw | CTTTCTTGGCTGCTGATACAACCgctCCTGCTTATAGCAGTGAAGTTC |
| oLC7119 | *CaARO1*R980A-SDM-Rv | GAACTTCACTGCTATAAGCAGGagcGGTTGTATCAGCAGCCAAGAAAG |
| oLC7120 | *CaARO1*E1072R-SDM-Fw | CAATTGGTGCTGATGCAGTTagaTTAAGAGTTGATTTATTTAAGG |
| oLC7121 | *CaARO1*E1072R-SDM-Rv | CCTTAAATAAATCAACTCTTAAtctAACTGCATCAGCACCAATTG |
| oLC7122 | *CaARO1*R1194E-SDM-Fw | CCATGAATGCTGATATTGTGgaaCTAGTCGGTAAGGCAAACTC |
| oLC7123 | *CaARO1*R1194E-SDM-Rv | GAGTTTGCCTTACCGACTAGttcCACAATATCAGCATTCATGG |
| oLC7124 | *CaARO1*K1334E-SDM-Fw | TAGCCATCACTATGCCATTGgaaTTGGATATCATGAAGTTTGC |
| oLC7125 | *CaARO1*K1334E-SDM-Rv | GCAAACTTCATGATATCCAAttcCAATGGCATAGTGATGGCTA |
| oLC7126 | *CaARO1*D1370A-SDM-Fw | GATATTTTGGTGATAACACTgctTGGGTGGGTATTAGTAATTC |
| oLC7127 | *CaARO1*D1370A-SDM-Rv | GAATTACTAATACCCACCCAagcAGTGTTATCACCAAAATATC |
| oLC7332 | SalI-stop-*CaARO1*-Rv for pBAD24 | cattgtcgacTTATTCCTCAACAACAGCAC |
| oLC7383 | *ARO1*homology-Rv | gtgatgatggtgatggtgcatGGTGGATCCGTAGCTTTTTGAATG |
| oLC7384 | *ARO1-*uphomol-*P_ACT1_* | GAATAAATTGAGAGTTTTTTGTTTCCCAACACCCCAATATCTAGGTATAACAGTTTATGAACAGCATAAGCTCGAGGAGCTATTAAGATC |
| oLC7385 | His_6_TEV-Fw | atgcaccatcaccatcatcacgaaaacc |
| oLC7387 | *ARG4*-*ARO1* | ggcgcgccattataagtaaatg |
| oLC7388 | *ARG4*-*ARO1*-Rv | TCTCAATTTATCTAGTTTAACATATAATTTCTTCTATTCAATCGACATACGTACACACGAATTCTCCTCTtcgatgaattcgagctcgtt |
| oLC7389 | ARO1-domain 2-delete-SDM-Fw | GATTTAAGTTTTGTGTTGggtagttcaggaagttcaggttcaagtggaagttcaggtagttcaggaagttcaAGTGATAAAAGTATCATT |
| oLC7390 | *ARO1*-domain 2-delete-SDM-Rv | AATGATACTTTTATCACTtgaacttcctgaactacctgaacttccacttgaacctgaacttcctgaactaccCAACACAAAACTTAAATC |
| oLC7391 | *ARO1*-domain 3-delete-SDM-Fw | AAATTTAAGATTGAGCTTggtagttcaggaagttcaggttcaagtggaagttcaggtagttcaggaagttcaGCTGGTAGATCAGCTGCT |
| oLC7392 | *ARO1*-domain 3-delete-SDM-Rv | AGCAGCTGATCTACCAGCtgaacttcctgaactacctgaacttccacttgaacctgaacttcctgaactaccAAGCTCAATCTTAAATTT |
| oLC7393 | *ARO1*-domain 4-delete-SDM-Fw | CTTATCACTGGTGCTGAAggtagttcaggaagttcaggttcaagtggaagttcaggtagttcaggaagttcaGCTAAAAAATTTTGGGTC |
| oLC7394 | *ARO1*-domain 4-delete-SDM-Rv | GACCCAAAATTTTTTAGCtgaacttcctgaactacctgaacttccacttgaacctgaacttcctgaactaccTTCAGCACCAGTGATAAG |
| oLC7395 | ARO1-SDM-domain5-SDM-delete-Fw | CGGCGAATTAAATCAAACTTATTTTGATtaaGGAGGTTTCACTGCTAAAAAATTTTGG |
| oLC7396 | *ARO1*-SDM-domain5-SDM-delete-Rv | CCAAAATTTTTTAGCAGTGAAACCTCCttaATCAAAATAAGTTTGATTTAATTCGCCG |
| oLC7409 | *CaARO1*W877K-SDM-Fw | GAAAATCTACTTTATCTGAAaaaTTGGCTTCCTTTTTGGGGTTC |
| oLC7410 | *CaARO1*W877K-SDM-Rv | GAACCCCAAAAAGGAAGCCAAtttTTCAGATAAAGTAGATTTTC |
| oLC7411 | *CaARO1*S880K-SDM-Fw | CTTTATCTGAATGGTTGGCTaaaTTTTTGGGGTTCAAGATGTTAG |
| oLC7412 | *CaARO1*S880K-SDM-Rv | CTAACATCTTGAACCCCAAAAAtttAGCCAACCATTCAGATAAAG |
| oLC7413 | *CaARO1*R1021E-SDM-Fw | GAGGATGAATTCAACCATTTGgaaAGATCCTTTGTAAACTACATTAAAC |
| oLC7414 | *CaARO1*R1021E-SDM-Rv | GTTTAATGTAGTTTACAAAGGATCTttcCAAATGGTTGAATTCATCCTC |
| oLC7415 | *CaARO1*R1022E-SDM-Fw | GATGAATTCAACCATTTGAGAgaaTCCTTTGTAAACTACATTAAAC |
| oLC7416 | *CaARO1*R1022E-SDM-Rv | GTTTAATGTAGTTTACAAAGGAttcTCTCAAATGGTTGAATTCATC |
| oLC7417 | *CaARO1*P1384EP1385E-SDM-Fw | CTTTCATAAGAGCAGGTGTTgaagaaAAGCTGAGCTCGAATGGGTTAG |
| oLC7418 | *CaARO1*P1384EP1385E-SDM-Rv | CTAACCCATTCGAGCTCAGCTTttcttcAACACCTGCTCTTATGAAAG |
| oLC7419 | *CaARO1*G1487E-SDM-Fw | CTAATGGAAGTGAACAATCTGCTgaaTTCAAGCCAACATTATTGG |
| oLC7420 | *CaARO1*G1487E-SDM-Rv | CCAATAATGTTGGCTTGAAttcAGCAGATTGTTCACTTCCATTAG |
| oLC7479 | SmaI-His_6_TEV-*ARO1*-Fw | cattCCCGGGatgcaccatcaccatcatcacgaaaacctctactttcaaggtATGTCTATTGAAAAGGTGCC |
| oLC7482 | *ARO1-ARG4* | catttacttataatggcgcgccTTATTCCTCAACAACAGCAC |
| oLC7601 | *CaARO1*seq1 | GTTACCAATTGAAAGCACATC |
| oLC7602 | *CaARO1*seq2 | GATAGATTTGGAACCAGGTGG |
| oLC7603 | *CaARO1*seq3 | CTGGTAAGACTTGGCCAGG |
| oLC7604 | *CaARO1*seq4 | TTTCCAGTGCCTCTCATACC |
| oLC7605 | *CaARO1*seq5 | GATGAATTCAACCATTTGAGAAG |
| oLC7606 | *CaARO1*seq6 | ACAACTTCATTCAAATCCGG |
| oLC7607 | *CaARO1*seq7 | TTAAATGGAACATTCACCCC |
| oLC7608 | *CaARO1*seq8 | CAAAATAAGTTTGATTTAATTCGCC |
| oLC8080 | frameshift-repair-minus-ClaI-Fw | CAAAACGTCATTATGTTGAAtTCGATATTTTGTTGAAgAAAATGGCCATTGACAAGAAAAATGATG |
| oLC8081 | frameshift-repair-minus-ClaI-Rv | CATCATTTTTCTTGTCAATGGCCATTTTcTTCAACAAAATATCAATTTCAACATAATGACGTTTTG |
| oLC8510 | *tetO-His_6_-TEV-CaARO1*-Rv | TACCCAAAATTGGCACCTTTTCAATAGAaccttgaaagtagaggttttcgtgatgatggtgatggtgcatcgactatttatatttgtatg |
| oLC8511 | *tetO-His_6_*-*CaARO1*-Rv | AACCAACATGGATAGTTTCCTTACCCAAAATTGGCACCTTTTCAATAGAgtgatgatggtgatggtgcatcgactatttatatttgtatg |
| oLC8966 | Fuse-Fw | GAATAAATTGAGAGTTTTTTGTTTCCCAAC |
| oLC8967 | Fuse-Rv | TCTCAATTTATCTAGTTTAACATATAATTTCTTCTATTC |
| oLC10k50 | *ARO1-*qPCR-Fw | ACAATTGGTGCTGATGCAGT |
| oLC10k51 | *ARO1-*qPCR-Rv | TGGGAATTTACCACCTTGCG |
| oLC10k52 | *DQD1*-qPCR-Fw | AGAGATGCCTTGTTGGGAAC |
| oLC10k53 | *DQD1*-qPCR-Rv | GCTGCCGTGTAACCATAAAC |
| oLC9710 | *P_DQD1_*-sgRNA | acttgcgtaaactatttttaatttgTTGTATGAAGTAGTGATGGAgttttagagctagaaatagcaagtt |
| oLC9711 | *tetO*-*CaDQD1*-Fw | CGGTCCGCTTACGTCGTGCTACGAAAAAAAAAAAGTCGAGAGTGAAAAATCATTCATTTAAGGTTGTTTCggaaacagctatgaccat |
| oLC9712 | *tetO*-*caDQD1*-Rv | ATTTTTCTGGCTCTCTGGTACCTAACAAATTAAGATTGGGACCATTAATGAGAAGTACTTTCTTAACCATcgactatttatatttgtatg |
| oLC9713 | *CaDQD1-*out-Fw | AGGTGTGTTCCATTCTTTCCAC |
| oLC9714 | *CaDQD1*-in-Rv | ATCACAACAAAACCGACACCC |
| oLC2285 | *ACT1*-qPCR-Fw | GACCTTGAGATACCCAATTG |
| oLC2286 | *ACT1-*qPCR-Rv | CAGCTTGAATGGAAACGTAG |
